# Mitochondrial dysfunction mediated through dynamin-related protein 1 (Drp1) propagates impairment in blood brain barrier in septic encephalopathy

**DOI:** 10.1186/s12974-019-1689-8

**Published:** 2020-01-27

**Authors:** Bereketeab Haileselassie, Amit U. Joshi, Paras S. Minhas, Riddhita Mukherjee, Katrin I. Andreasson, Daria Mochly-Rosen

**Affiliations:** 1 Department of Chemical and Systems Biology, Stanford University School of Medicine, Stanford, CA USA; 2 Department of Pediatrics, Stanford University School of Medicine, Stanford, CA USA; 3 Department of Neurology & Neurological Sciences, Stanford School of Medicine, Stanford, CA USA

**Keywords:** Drp1, Neuroinflammation, Blood brain barrier, P110, Sepsis

## Abstract

**Background:**

Out of the myriad of complications associated with septic shock, septic-associated encephalopathy (SAE) carries a significant risk of morbidity and mortality. Blood-brain-barrier (BBB) impairment, which subsequently leads to increased vascular permeability, has been associated with neuronal injury in sepsis. Thus, preventing BBB damage is an attractive therapeutic target. Mitochondrial dysfunction is an important contributor of sepsis-induced multi-organ system failure. More recently, mitochondrial dysfunction in endothelial cells has been implicated in mediating BBB failure in stroke, multiple sclerosis and in other neuroinflammatory disorders. Here, we focused on Drp1-mediated mitochondrial dysfunction in endothelial cells as a potential target to prevent BBB failure in sepsis.

**Methods:**

We used lipopolysaccharide (LPS) to induce inflammation and BBB disruption in a cell culture as well as in murine model of sepsis. BBB disruption was assessed by measuring levels of key tight-junction proteins. Brain cytokines levels, oxidative stress markers, and activity of mitochondrial complexes were measured using biochemical assays. Astrocyte and microglial activation were measured using immunoblotting and qPCR. Transwell cultures of brain microvascular endothelial cells co-cultured with astrocytes were used to assess the effect of LPS on expression of tight-junction proteins, mitochondrial function, and permeability to fluorescein isothiocyanate (FITC) dextran. Finally, primary neuronal cultures exposed to LPS were assessed for mitochondrial dysfunction.

**Results:**

LPS induced a strong brain inflammatory response and oxidative stress in mice which was associated with increased Drp1 activation and mitochondrial localization. Particularly, Drp1-(Fission 1) Fis1-mediated oxidative stress also led to an increase in expression of vascular permeability regulators in the septic mice. Similarly, mitochondrial defects mediated via Drp1-Fis1 interaction in primary microvascular endothelial cells were associated with increased BBB permeability and loss of tight-junctions after acute LPS injury. P110, an inhibitor of Drp1-Fis1 interaction, abrogated these defects, thus indicating a critical role for this interaction in mediating sepsis-induced brain dysfunction. Finally, LPS mediated a direct toxic effect on primary cortical neurons, which was abolished by P110 treatment.

**Conclusions:**

LPS-induced impairment of BBB appears to be dependent on Drp1-Fis1-mediated mitochondrial dysfunction. Inhibition of mitochondrial dysfunction with P110 may have potential therapeutic significance in septic encephalopathy.

## Background

Sepsis, an exaggerated systemic inflammatory response syndrome, has often been associated with multi-organ damage and subsequent increased long-term morbidity and mortality [1, 2]. Sepsis-associated encephalopathy (SAE) is a common but poorly understood neurological complication of sepsis that is often associated with cerebral coagulopathy, ischemia, and inflammation in the brain. Clinically, SAE is linked with a high risk of mortality, and survivors often suffer from chronic autonomic nerve dysfunctions, delirium, or damaged cognitive functions of different degrees [3]. Despite the prevalence and severity of this condition, there is no prophylactic or targeted therapy to abrogate neurologic injury in septic patients, and standard of care is limited to the non-specific supportive measures and treatment of the underlying infection [4, 5].

While the pathobiology behind septic encephalopathy is still poorly understood, many mechanisms have been proposed [6, 7]. The current hypothesis suggests that a combination of dysregulated brain perfusion and oxygenation resulting in altered blood-brain-barrier (BBB) contributes significantly to the neurologic dysfunction in sepsis [4, 8–10]. This phenomenon is further enhanced by other contributors, such as renal and/or hepatic dysfunction, disglycemia, fever, and the use of neurotropic drugs, as well as environmental factors [11].

More recently, a focus on “cytopathic hypoxia,” a disturbance of cellular bioenergetics in sepsis, has revealed the influence of mitochondrial damage on end-organ failure and eventual mortality [11–14]. Furthermore, the role of mitochondrial failure is associated with dysfunctional BBB in other neurologic disorders, including stroke, Alzheimer’s disease (AD), Parkinson disease (PD), Huntington’s disease, and epilepsy [15–19]. Despite these findings, the role of altered mitochondrial dynamics (fission, fusion, and mitophagy*)*, which is key for mitochondrial quality control [20], has not been adequately explored in the context of BBB dysfunction and subsequent septic encephalopathy.

We have previously demonstrated a therapeutic effect of peptide P110, a 7-amino acid peptide representing a homology sequence between dynamin-related protein 1 (Drp1) and Fission 1 (Fis1) [21], in reducing pathological mitochondrial dysfunction in multiple neurodegenerative diseases [20, 22–25]. This project evaluates the role of Drp1/Fis1 interaction in mediating mitochondrial failure and subsequent BBB dysfunction in the setting of sepsis.

## Methods

### Animals

All animal experimental procedures were conducted in accordance with the animal care regulations of the National Institute of Health and were approved by Stanford University’s Administrative Panel on Laboratory Animal Care. The mice were maintained on a 12-h light/dark cycle in stable conditions in terms of temperature, humidity, and ventilation. Water and food were offered ad libitum. Animals were randomly assigned numbers and evaluated thereafter by a researcher blinded to both experimental condition and genotype.

#### In vivo model for severe septic injury

Five to seven-week-old BALB/c mice were randomized into control (*n* = 4), lipopolysaccharide (LPS) (*n* = 8), and LPS + P110-treated (*n* = 8) groups. LPS was administered at 8 mg/kg to induce a severe sepsis phenotype [26]. Peptide P110 was administered at 0.5 mg/kg/day (dissolved in 0.2 ml of saline) intraperitoneally, 3 hrs following LPS.

### Primary brain microvascular endothelial cells

Primary brain microvascular endothelial cells (BMECs) were isolated from adult mice according to existing protocols [27]. In brief, BMECs were cultured in Dulbecco’s modified Eagle’s medium (DMEM)/F12 supplemented with 20% FBS, 1 % GlutaMAX (Life Technologies), basic fibroblast growth factor (BFGF; 1 ng/ml; Roche Life Sciences), heparin (100 μg/ml), insulin (5 μg/ml), transferrin (5 μg/ml), selenium (5 ng/ml) (insulin-transferrin-selenium medium supplement; Life Technologies), and gentamicin (50 μg/ml; Sigma Aldrich). Puromycin (4 μg/ml; Sigma Aldrich) was added to the BMEC medium for the first 48 h after plating to remove pericytes and increase endothelial cell purity. Cultures were maintained at 37 °C in a humidified atmosphere of 5% CO2/95% air. The purified primary BMECs were used to construct in vitro BBB model when 80% confluent.

### Primary astrocyte culture

Primary astrocyte cultures were prepared from cerebral cortices of P0/P1 pups (C57BL/6). In brief, the meninges were carefully removed from cortices using a dissecting microscope. Then, the cleaned cortices were mechanically dissociated in astrocyte culture medium. Astrocytes were grown on poly-l-lysine-coated 24-well plates in high glucose DMEM (Life Technologies) supplemented with 10% fetal bovine serum [25].

### Construction of in vitro BBB model

BMECs were co-cultured with astrocytes [27]. In brief, BMECs were seeded on the inside of the Transwell insert, which was coated with fibronectin and collagen type IV (0.1 mg/ml, each), at a density of 4 × 10^4^ cells per well. The inserts were then placed in a 24-well plate containing primary astrocytes. The medium used to plate the cells in the 24-well Transwell plate. The medium in the luminal chamber (or the inside of the Transwell insert) was changed 24 h after seeding. BMEC monolayers were cultured for 3 days before use in experiments.

### Primary neuron culture

Primary neuron cultures were prepared from cerebral cortices of embryonic day E17 WT (C57BL/6) [25]. In brief, cortices were dissected and dissociated using papain dissociation system (Worthington Biochemical Corporation). Cells were cultured at 20,000/ well of a 96-well plate coated with poly-d-lysine (Sigma) for cell viability assays. For seahorse experiments, 1 × 10^5^ cells/well were seeded in XF 24-well cell culture microplate and cultured in Neurobasal medium (Invitrogen) supplemented with B-27 (Invitrogen) containing 25 mM glucose, 4 mM glutamine, 1 mM sodium pyruvate, and 5% FBS. At 24 h after seeding, the medium was changed to Neurobasal medium supplemented with B-27 and 0.5 mM glutamine. Cells were cultured at 37 °C in a humidified chamber of 95% air and 5% CO_2_. Cultures were used for experiments from 7 to 10 days after seeding.

### Trans-endothelial barrier permeability

Three hours after the treatment with LPS, fluorescein isothiocyanate (FITC)-tagged 150-kDa dextran (Sigma) was added to the upper (luminal) chamber at a final concentration of 0.5 mg/ml as previously described [28]. Relative fluorescence (485-nm excitation/535-nm emission) was determined at the end of the 24-h incubation period by collection of 100-μl aliquots in triplicate from each lower (abluminal) chamber and measurement of fluorescence using SpectraMax M2e (Molecular devices),

### Cell and mitochondrial function assays

#### Mitochondrial membrane potential

Cells were incubated with tetra-methyl-rhodamine methyl ester (200 nM; TMRM, Invitrogen) in HBSS (Hank’s balanced salt solution) for 30 min at 37 °C, as per the manufacturer’s protocol, and the fluorescence was analyzed using SpectraMax M2e (Molecular devices, using excitation at 360 nm and emission at 460 nm).

#### ATP measurements

Relative intracellular ATP levels were determined using ATP-based Cell Titer-Glo Luminescent Cell Viability kit (Promega), which causes cell lysis and generates a luminescent signal proportional to the amount of ATP present. In brief for intracellular ATP levels, opaque-walled 96-well plates with cell lysate (50 μl) were prepared. An equal volume of the single-one-step reagent provided by the kit was added to each well and incubated for 30 min at room temperature. ATP content was measured using a luminescent plate reader SpectraMax M2e (Molecular devices).

#### ROS production

For cellular ROS detection, cells were incubated with 2,7 dichloro-fluorescein diacetate (DCFDA) (Abcam) 100 μM for 30 min at 37 °C in the dark, and fluorescence was analyzed with excitation/emission at 495/529 nm, using SpectraMax M2e (Molecular devices). Fluorescence intensity was then normalized for cell number. To determine mitochondrial ROS production, cells were treated with 5 μM MitoSOX^TM^ Red, a mitochondrial superoxide indicator (Invitrogen) for 10 min at 37 °C, according to the manufacturer’s protocol, and fluorescence was analyzed with excitation/emission at 510/580 nm, using SpectraMax M2e (Molecular devices).

#### Bioenergetic profiles

Cells were plated in a Seahorse XF24 Cell Culture Microplate (Agilent). All seahorse experiments in neurons were performed at 24 h after individual stimuli. At the end of the treatment, cells were washed twice with Agilent Seahorse XF Media (Agilent) supplemented with 1 mM pyruvate, 2 mM l-glutamine, and 2 mM d-glucose; a final volume of 525 μl was placed in each well. Cells were then incubated in a 0% CO_2_ chamber at 37 °C for 1 h before being placed into a Seahorse XFe24 Analyzer (Agilent). For oxygen consumption rate (OCR) and extracellular acidification rate (ECAR) experiments, cells were treated with 1 μM oligomycin, 2 μM carbonyl cyanide p-trifluoromethoxy phenylhydrazone (FCCP), and 0.5 μM rotenone/antimycin. A total of three OCR and pH measurements were taken after each compound was administered. All seahorse experiments were repeated at least three times.

#### Cell death

Cytotoxicity was determined using Cytotoxicity Detection Kit, as before [23]. In brief, media was collected at endpoints (in phenol red-free DMEM) to measure the percentage of released lactate dehydrogenase activity (LDH). To quantify total LDH, cells were lysed with Triton X (1% in serum-free cell culture media) overnight at 4 °C; 50 μl media or lysate was transferred with 50 μl of reaction mix in a 96-well plate and incubated at RT for 30 min in the dark. Absorbance was measured at 490 nm using SpectraMax M2e (Molecular devices), and cell death is presented as a percent of released LDH of total LDH.

#### Caspase activity assay

Caspase 3 activities were determined using a colorimetric caspase 3 assay kit (Abcam), according to the manufacturer’s protocol. Cell lysates containing 200 μg protein were used for each assay. Each assay was repeated with three independent cell cultures. Absorbance at 400 nm was recorded using SpectraMax M2e (Molecular devices). Caspase activity was calculated in arbitrary units and represented as fold change of WT control.

### Western blot analysis

Protein concentrations were determined using the Bradford assay (Thermo Fisher Scientific). Proteins were resuspended in Laemmli buffer containing 2-mercaptoethanol, loaded on SDS–PAGE, and transferred on to nitrocellulose membrane, 0.45 μm (Bio-Rad), as before [20]. Cell supernatant was cleared of cellular debris by centrifugation at 1000*g* for 10 min. The total lysate was resuspended in Laemmli buffer containing 2-mercaptoethanol, loaded on SDS–PAGE, and transferred on to nitrocellulose membrane, 0.45 μm (Bio-Rad), as before [20]. Membranes were cut at appropriate molecular weights and then probed with the indicated antibody and visualized by ECL (0.225 mM p-coumaric acid; Sigma), 1.25 mM 3-aminophthalhydrazide (Luminol; Fluka) in 1 M Tris pH 8.5. Scanned images of the exposed X-ray film or images acquired with Azure Biosystems C600 were analyzed with ImageJ to determine relative band intensity. Quantification was performed on samples from independent cultures for each condition.

### RNA isolation and gene expression analysis

RNA isolation was performed using GenElute™ Mammalian Total RNA Miniprep Kit (Sigma Aldrich) according to the manufacturer’s protocols. RNA concentration was measured using a Nanodrop (ND −1000; NanoDrop Technologies, Rockland, DE, USA), and RNA integrity was assessed using a Bioanalyzer (2100; Agilent Technologies, Palo Alto, CA, USA). cDNA synthesis was performed using the Quantitect reverse transcription kit (Qiagen) according to the manufacturer’s instructions, with a minimal input of 200 ng total RNA. Quantitative PCR (qPCR) was performed using the 7300 Real Time PCR system (Applied Biosystems, Foster City, USA) using the equivalent cDNA amount of 1–2 ng total RNA used in cDNA synthesis. SYBRgreen master mix (Applied Biosystems) and a 2 pmol/ml mix of forward and reverse primer sequences were used for 40 cycles of target gene amplification.

### Statistical analysis

Prism 8.0 (GraphPad Software) was used for the statistical analysis. Data shown are the mean ± SD. with *P* < 0.05 considered statistically significant. Group differences were analyzed with one-way analysis of variance (ANOVA) followed by Holms-Sidak multiple comparisons test for multiple groups. Data distribution was assumed to be normal, but this was not formally tested. No statistical methods were used to predetermine sample sizes.

## Results

### Drp1-Fis1-mediated mitochondrial dysfunction is a key mechanism in LPS-induced brain microvascular permeability

**Fig. 1:**
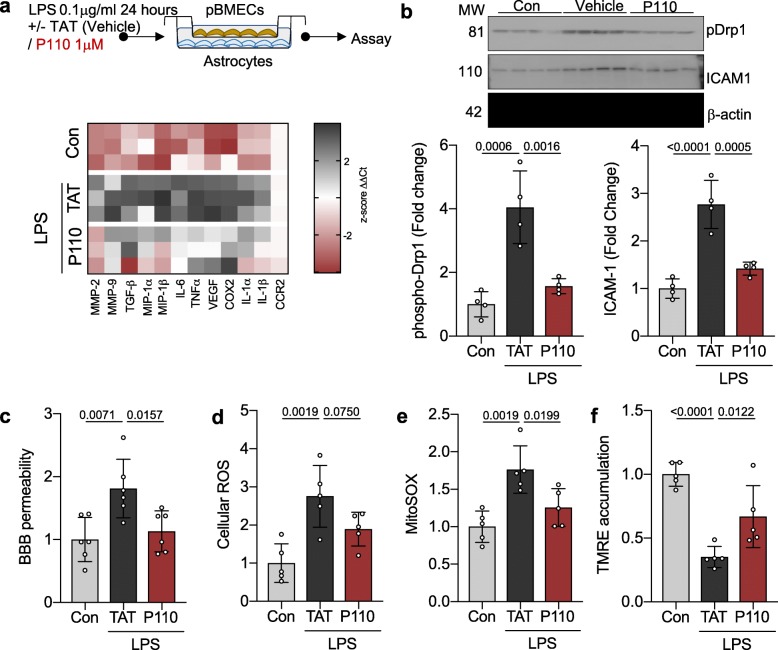
Drp1-Fis1-mediated mitochondrial dysfunction is a key mechanism for LPS-induced brain microvascular permeability. **a** Primary brain microvascular endothelial cells co-cultured with astrocytes were treated with 0.1 mg/ml LPS in the presence or absence of P110 (1 mM) for 24 h (*n* = 3). Gene expression of vascular integrity modifiers was measured by real-time PCR and represented as z-score. **b** phospho Drp1 (Ser 616) levels and ICAM-1 levels were quantified by immunoblotting and represented as fold change of control. β-actin was used as a loading control (*n* = 4). **c** Blood-brain-barrier integrity were assessed by measuring concomitant paracellular permeability (luminal to abluminal) to labeled dextran (150 kDa) after treatment as in **a** and represented as fold change of control (*n* = 6). **d** Cellular ROS was measured using CM-H2DCFDA (General Oxidative Stress Indicator) after treatment as in **a** and represented as fold change of control (*n* = 5). **e** Mitochondrial ROS was measured using MitoSOX^TM^ after treatment as in **a** and represented as fold change of control (*n* = 5). **f** Mitochondrial membrane potential was measured using TMRE after treatment as in **a** and represented as fold change of control (*n* = 5). Probability determined by one-way ANOVA and Holm-Sidak’s test for multiple comparisons between each treatment group, as above. All data are shown as the mean ± s.d., and *p* values are indicated

Gene expression profile of vascular integrity and inflammation of primary brain microvascular endothelial cells co-cultured with astrocytes demonstrates a significant shift to a pro-inflammatory phenotype and activation of key mediators of vascular endothelial permeability following LPS treatment (0.1 μg/ml for 24 hrs) (Fig. 1a). This is associated with pathologic mitochondrial Drp1 activation, as measured by phosphorylation at Serine 616 (fold change 4.05 ± 1.142, *p* = 0.0006), suggesting a shift towards a pro-fission state [20, 23, 24, 26] (Fig. 1b). The mitochondrial damage in microvascular endothelial cells and loss of BBB integrity is correlated with increased mitochondrial specific (MitoSOX; *p* = 0.002) as well as total (*p* = 0.002) oxidative stress as well as a loss of mitochondrial membrane potential (TMRE; *p* < 0.001) following LPS treatment (Fig. 1c–f).

LPS-mediated derangement in vascular endothelial function were ameliorated with P110 treatment, represented by limited activation of key enzymes that mediate vascular endothelial permeability as well as limited Drp1 activation and mitochondrial localization. P110 treatment also lead to improvement in BBB integrity and improved mitochondrial health represented by limited oxidative stress (MitoSox; *p* = 0.02) as well as improved mitochondrial membrane potential (TMRM; *p* = 0.01) (Fig. 1).

Inhibition of Drp1-Fis1-mediated mitochondrial dysfunction is protective against LPS-mediated neuronal injury in culture

**Fig. 2:**
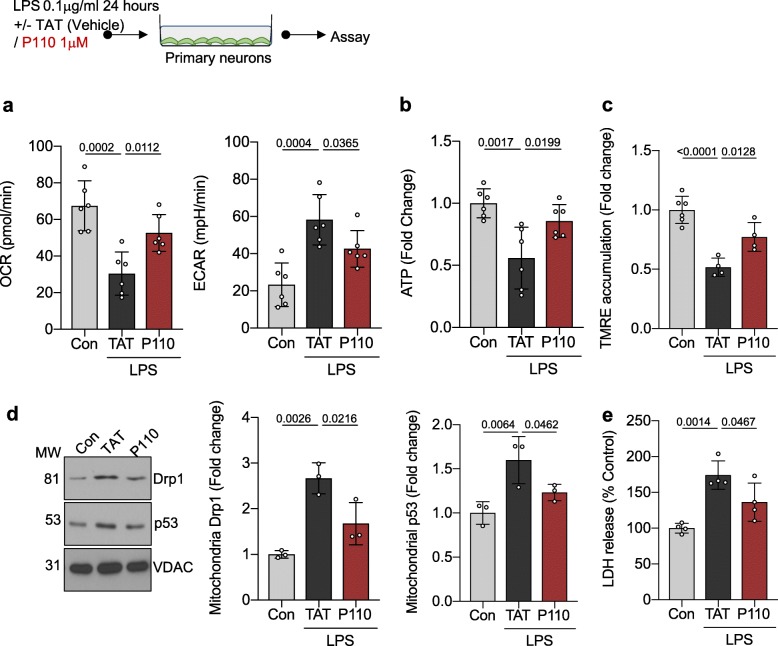
Inhibition of Drp1-Fis1-mediated mitochondrial dysfunction is protective against LPS-mediated neuronal injury in culture. **a** Oxidative phosphorylation and glycolytic rate was measured with Seahorse Extracellular Flux in primary neurons were treated with 0.1 mg/ml LPS in the presence/absence of P110 (1 mM) for 24 h (*n* = 6). **b** Cellular ATP level was measured using CellTiter-Glo® after treatment as in **a** and represented as fold change of control. **c** Mitochondrial membrane potential was measured using TMRE after treatment as in **a** and represented as fold change of control (*n* = 6). **d** Drp1 levels and p53 levels were quantified by immunoblotting in enriched mitochondrial fractions and represented as fold change of control. VDAC was used as a loading control (*n* = 3). **e** LDH release was measured after treatment as in **a** and represented as % of Control (Con) (*n* = 6). Probability determined by one-way ANOVA and Holm-Sidak’s test for multiple comparisons between each treatment group, as above. All data are shown as the mean ± s.d., and *p* values are indicated

When evaluating the primary neurons, LPS treatment caused a significant mitochondrial damage, leading to a decrease in oxidative phosphorylation (basal OCR; *p* = 0.0002) and subsequent shift towards glycolysis (basal ECAR; *p* = 0.0004). Mitochondrial damage was further supported by the decreased mitochondrial membrane potential (TMRE; *p* = < 0.0001) and ATP production (fold change of control = 0.557 ± 0.002, *p* = 0.0017) following LPS treatment (Fig. 2a–c). Similar to the brain microvascular endothelial cells, LPS-treated primary neurons had an increase in Drp1 activation and mitochondrial localization (fold change 1.86 ± 0.31, *p* = 0.003), suggesting a role for Drp1 in the progression of mitochondrial failure in the setting of sepsis. LPS treatment of primary neurons subsequently led to increased p53 association with the mitochondrial membrane (fold change = 1.59 ± 0.26, *p* = 0.0064) and increased cell death (LDH (% of control); *p* = 0.001).

Further supporting the role of Drp1-Fis1-mediated derangements in mitochondrial dysfunction under LPS stimulation, peptide P110-treated neurons had improved mitochondrial function (basal OCR; *p* = 0.0112) and subsequent decreased glycolysis (basal ECAR; *p* = 0.0365) leading to improved ATP production (fold change of control = 0.857 ± 0.13, *p* = 0.0199). P110 treatment was also noted to abrogate the downstream effects of LPS treatment by limiting p53 activation (mitochondrial p53 fold change 1.23 ± 0.09, *p* = 0.0462) and subsequent cell death (LDH (% of control); *p* = 0.0467).

Inhibition of Drp1/Fis1-mediated mitochondrial dysfunction is protective in a mouse model of septic encephalopathy

**Fig. 3:**
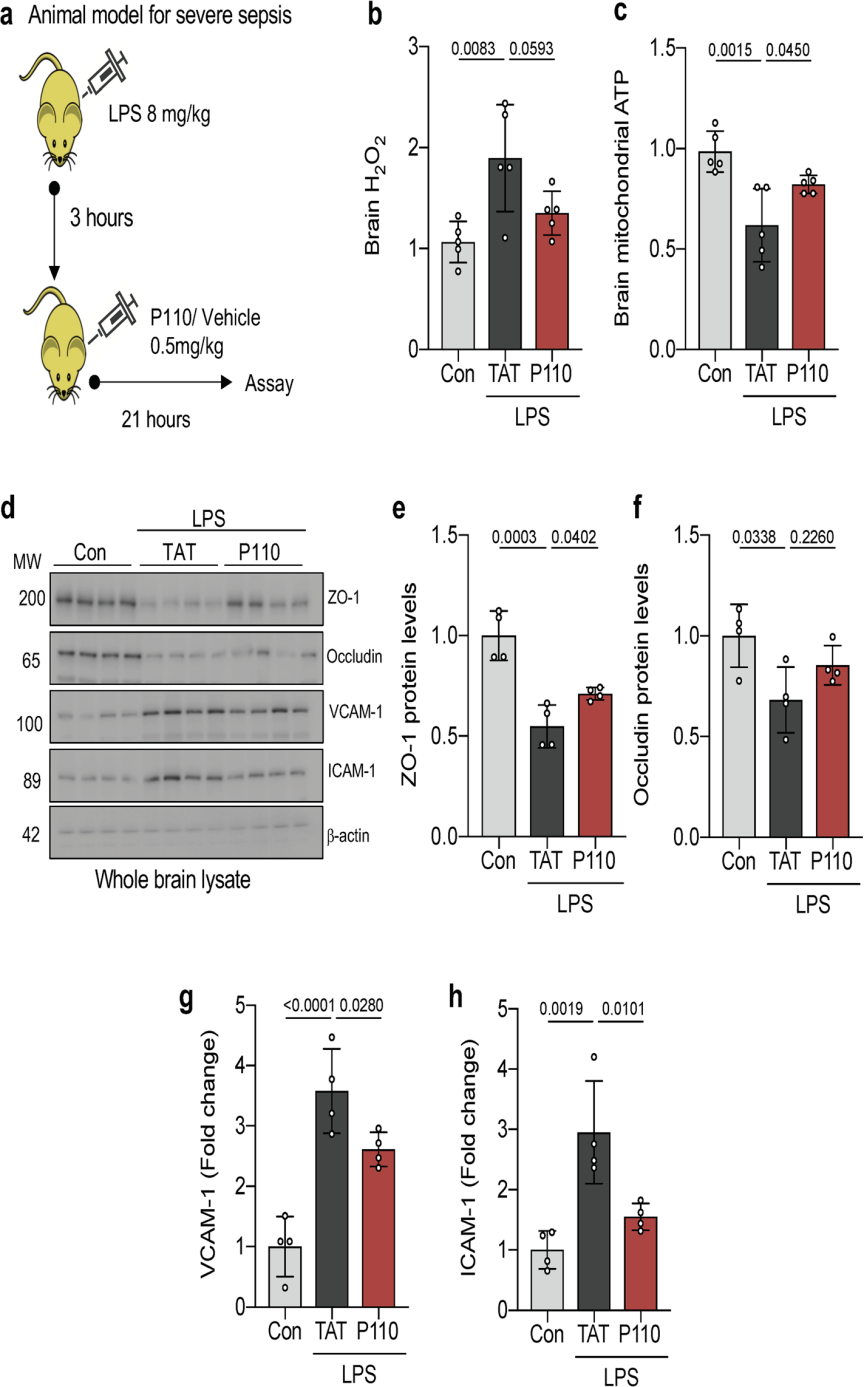
Inhibition of Drp1-Fis1-mediated mitochondrial dysfunction reduces oxidative stress in the brain and preserves endothelial integrity. **a** Balb/c mice were treated with LPS (8 mg/kg IP) to induce murine model of sepsis. Peptide P110 (0.5 mg/kg IP) was administered in a subset of septic animals 3 h following LPS treatment and tissue harvested at 24 h. **b** Hydrogen peroxide production was determined by Amplex Red^TM^ from whole brain mitochondria after treatment as in **a** (*n* = 4). **c** ATP colorimetric assay kit was used to measure ATP levels in freshly isolated whole brain mitochondria after treatment as in **a** (*n* = 4). **d** ZO-1, occludin, VCAM-1, and ICAM-1 levels were quantified by immunoblotting. β-actin was used as a loading control (*n* = 4). **e** ZO-1, **f** occludin, **g** VCAM-1, and **h** ICAM-1 protein levels were quantified and represented as fold change (*n* = 4). Probability determined by one-way ANOVA and Holm-Sidak’s test for multiple comparisons between each treatment group, as above. All data are shown as the mean ± s.d., and *p* values are indicated

Using an established LPS model of sepsis (28) in 5 to 7-week-old female BALB/c mice, we evaluated the role of Drp1/Fis1 interaction in sepsis-mediated encephalopathy. Brain tissue from septic mice demonstrated a significant decrease in mitochondrial function, represented by increased oxidative stress (brain H_2_O_2_; *p* = 0.01) as well as decreased ATP levels (*p* = 0.008), which was significantly improved with P110 treatment (Fig. 3b, c). When evaluating BBB integrity, LPS treatment caused a significant decrease in proteins forming the vascular tight junctions (ZO-1 and occludin) and upregulation of vascular leukocyte adhesion molecules (VCAM-1 and ICAM-1), suggesting a compromise to BBB integrity which was abrogated under P110 treatment (Fig. 3d–h).

**Fig. 4:**
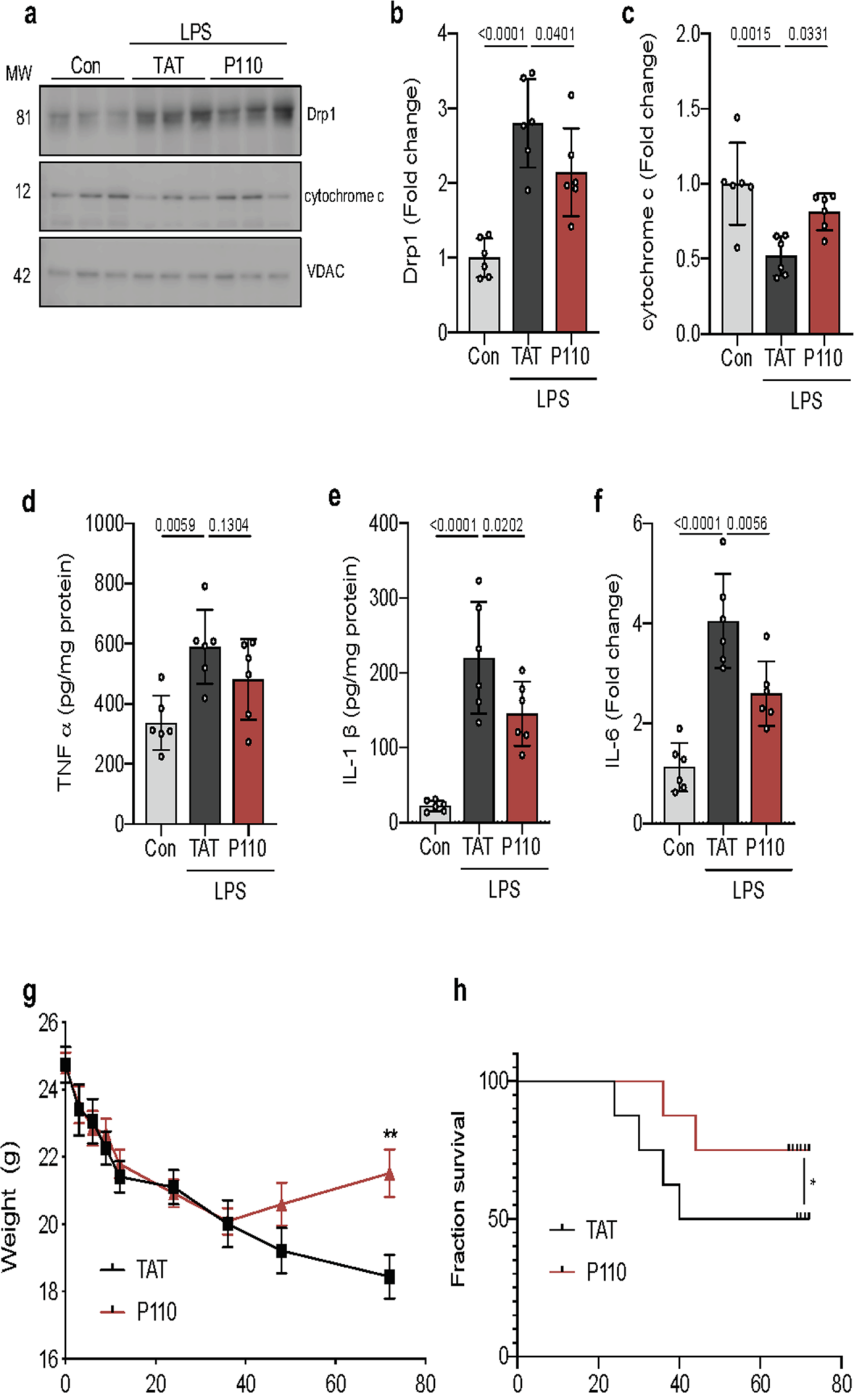
Inhibition of Drp1-Fis1-mediated mitochondrial dysfunction is protective in mouse model of septic encephalopathy. **a** Mitochondrial Drp1 translocation and cytochrome c release were quantified by immunoblotting in enriched mitochondrial fractions. VDAC was used as a loading control. **b** Drp1 and **c** cytochrome c protein levels were quantified and represented as fold change (*n* = 6). **d** TNFα, **e** IL-1β, and **f** IL-6 cytokine levels were measured in whole brain lysate by ELISA after a 24-h treatment of LPS (*n* = 6). **g** Weight changes in these mice were quantified as a surrogate for illness severity. (*n* = 5 mice for control, 5 for LPS-treated mice, and 5 for LPS + P110-treated). **h** Kaplan–Meier survival curve of mice showing increased survival following P110 treatment (red trace) as compared to the trans-activator of transcription (TAT)-treated control mice (black trace). Probability determined by one-way ANOVA and Holm-Sidak’s test for multiple comparisons between each treatment group, as above. All data are shown as the mean ± s.d., and *p* values are indicated

Similar to the findings using cell culture model, LPS-mediated derangements in mitochondrial function in brain tissue of septic mice was associated with Drp1 activation, represented by increased Drp1 phosphorylation and mitochondrial localization (fold change 2.801 ± 0.59; *p* < 0.001). These increases correlated with activation of cell death pathways, including leak of mitochondrial cytochrome c (fold change 0.52 ± 0.13, *p* = 0.0015), a potent apoptotic trigger (Fig. 4a–c). Inhibition of Drp1/Fis1 interaction, using P110, reduced the level of released mitochondrial cytochrome C (fold change, LPS = 0.52 ± 0.13 vs LPS + p110 = 0.81 ± 0.12, *p* = 0.33) and decreased the level of neuroinflammatory markers, TNFα, ILI-β, and IL-6, seen in septic mice (Fig. 4d–f).

When evaluating morbidity and mortality in this murine model of sepsis, P110 treatment reduced clinical disease severity, evidenced by significantly reduced weight loss and a faster weight recovery (Fig. 4g). Importantly, P110 treatment reduced mortality by 50% at 48 h (which was sustained through 94 h) relative to LPS treatment alone (Fig. 4h).

## Discussion

Sepsis-associated encephalopathy (SAE) is a prevalent complication of sepsis. Currently, it is thought to affect over 75% patients with sepsis [7, 12] and causes a higher risk of mortality in those who are afflicted. Additionally, the long-term sequelae for those who survive (which include cognitive impairment, hippocampal atrophy, white matter disease, and ischemic strokes) confer significant limitations on quality of life and incurs a staggering healthcare cost [1, 3, 9, 29].

The pathophysiology of SAE is complex and involves brain microvascular cell dysfunction, loss of blood-brain barrier integrity, microglia and astrocytic activation, and neuronal death [3]. However, not much is known about the role of mitochondrial health in the pathogenesis of sepsis. In the present study, we demonstrate that Drp1 activation and subsequent mitochondrial dysfunction plays a critical role in propagating LPS-mediated SAE using cell culture models of blood brain barrier as well as a mouse model of severe sepsis. In particular, we demonstrate that selective inhibition of Drp1/Fis1 interaction, using P110, reduces BBB dysfunction and improves neuronal health in culture models. Furthermore, treatment with P110 3 h after LPS injection improved mitochondrial health and reduced inflammation in the brain, and improved survival in a mouse model of severe sepsis.

The role of mitochondrial health as a mechanistic link between inflammation and cellular damage in neuroinflammatory and neurodegenerative diseases has become increasingly recognized. In stroke models, LPS-induced derangement in mitochondrial health exacerbates infarct volume and opens BBB in mice subjected to a transient occlusion of the mid carotid artery [15]. Furthermore, LPS increases the paracellular permeability of brain endothelial cells and decreases trans-endothelial electrical resistance [30]. Brain endothelial cells lining the cerebral micro-vessels constitute a physical barrier between blood and brain tissue by a complex network of tight junctions consisting occludin, claudins, junctional adhesion molecules (JAMs), and zonula occludens (ZO)-1, 2, and 3 [31]. Of these proteins, ZO-1 is critical in maintaining the junction assembly and its absence has been shown to correlate with BBB failure [32]. Additionally, alteration of the vascular endothelial permeability is thought to result from the release of cytokines, free radicals, matrix metalloproteinases, and nitric oxide [33].

In the in vitro BBB model, we observed that LPS treatment robustly upregulated the gene expression of several pro-inflammatory markers, IL-6, TNFα, IL-1α, and IL1-β as well as vascular modifiers MMP-2, MMP-9, and TGF-β [33]. These changes are associated with alterations in mitochondrial dynamics as evidenced by temporal association with Drp1 phosphorylation (activation) and mitochondrial fragmentation. Furthermore, the reversal of LPS-mediated changes in proteins that modify vascular permeability and subsequent preservation of BBB integrity in the presence of P110 further supports the causal association between mitochondrial health and brain microvascular endothelial cell function.

Our earlier studies indicated a protective effect of P110 in multiple neuronal cell models [20, 21, 23]. Similarly, in the present study, we demonstrate a beneficial effect of blocking Drp1/Fis1 interaction on primary neurons exposed to LPS. Mitochondria are critical to many neuronal functions, and any alterations in their health have deleterious effects [34]. LPS interferes with mitochondrial transcription and oxidative phosphorylation (OXPHOS) [35]. Similarly, we observed a decrease in cellular respiration and a shift towards glycolysis under LPS stimulation. However, this metabolic shift did not compensate for the ATP production, subsequently leading to further loss of mitochondrial membrane potential, propagation of Drp1 and p53 recruitment to the mitochondrial outer membrane, and initiation of cell death pathways. All these effects are greatly blunted by P110 treatment as demonstrated by increased ATP levels as well as by improved cell survival.

Similar to the cell culture model used here, injection of a high dose of LPS resulted in an overall decrease in mitochondrial ATP as well as increased ROS levels in the mouse brain. Additionally, the observed Drp1/Fis1-dependent effect on intercellular adhesion molecule-1 (ICAM-1) and vascular cell adhesion molecule-1 (VCAM-1) suggests a connection between mitochondrial function and inflammatory cell adherence/transmigration across the BBB [36]. Impotently, LPS-mediated changes in brain mitochondrial function were greatly reduced by P110 treatment, as demonstrated by abrogation in brain vascular endothelial dysfunction, improvement in mouse illness severity (represented by weight loss), and survival.

## Conclusion

In conclusion, we demonstrate Drp1/Fis1 interaction as the mechanism responsible for SAE. Our study identified a potentially new therapeutic strategy to reduce the substantial risk of SAE-associated mortality and morbidity. Future large animal and clinical studies are necessary to evaluate the significance of this mechanistic pathway and therapeutic target in abrogating neuronal injury in the setting of sepsis.

## Data Availability

The datasets used and/or analyzed during the current study are available from the corresponding author on reasonable request.

## Abbreviations

AD: Alzheimer’s disease
ANOVA: Analysis of variance
BBB: Blood-brain-barrier
BFGF: Basic fibroblast growth factor
BMEC: Brain microvascular endothelial cells
DCFDA: Dichloro-fluorescein diacetate
DMEM: Dulbecco’s modified Eagle’s medium
Drp1: Dynamin-related protein 1
ECAR: Extracellular acidification rate
Fis1: Fission 1
FITC: Fluorescein isothiocyanate
ICAM-1: Intercellular adhesion molecule-1
JAMs: Junctional adhesion molecules
LDH: Lactate dehydrogenase activity
LPS: Lipopolysaccharide
OCR: Oxygen consumption rate
PD: Parkinson disease
qPCR: Quantitative PCR
SAE: Sepsis-associated encephalopathy
TAT: Trans-activator of transcription
TMRM: Tetra-methyl-rhodamine methyl ester
VCAM-1: Vascular cell adhesion molecule-1
ZO: Zonula occludens
